# Epithelial cell senescence induces pulmonary fibrosis through Nanog-mediated fibroblast activation

**DOI:** 10.18632/aging.102613

**Published:** 2019-12-31

**Authors:** Xiang Chen, Hongyang Xu, Jiwei Hou, Hui Wang, Yi Zheng, Hui Li, Hourong Cai, Xiaodong Han, Jinghong Dai

**Affiliations:** 1 Department of Pulmonary and Critical Care Medicine, The Affiliated Drum Tower Hospital of Nanjing University Medical School, Nanjing 210008, China; 2 Immunology and Reproduction Biology Laboratory and State Key Laboratory of Analytical Chemistry for Life Science, Medical School, Nanjing University, Nanjing 210093, China; 3 Jiangsu Key Laboratory of Molecular Medicine, Nanjing 210093, China; 4 Department of Critical Care Medicine, The Affiliated WuXi People's Hospital of Nanjing Medical University, Wuxi 214023, China

**Keywords:** idiopathic pulmonary fibrosis (IPF), epithelial cell senescence, pulmonary fibroblast activation, wnt/β-catenin signalling, nanog

## Abstract

Idiopathic pulmonary fibrosis (IPF) is a chronic and progressive lung disease tightly correlated with aging. The pathological features of IPF include epithelial cell senescence and abundant foci of highly activated pulmonary fibroblasts. However, the underlying mechanism between epithelial cell senescence and pulmonary fibroblast activation remain to be elucidated. In our study, we demonstrated that Nanog, as a pluripotency gene, played an essential role in the activation of pulmonary fibroblasts. In the progression of IPF, senescent epithelial cells could contribute to the activation of pulmonary fibroblasts via increasing the expression of senescence-associated secretory phenotype (SASP). In addition, we found activated pulmonary fibroblasts exhibited aberrant activation of Wnt/β-catenin signalling and elevated expression of Nanog. Further study revealed that the activation of Wnt/β-catenin signalling was responsible for senescent epithelial cell-induced Nanog phenotype in pulmonary fibroblasts. β-catenin was observed to bind to the promoter of Nanog during the activation of pulmonary fibroblasts. Targeted inhibition of epithelial cell senescence or Nanog could effectively suppress the activation of pulmonary fibroblasts and impair the development of pulmonary fibrosis, indicating a potential for the exploration of novel anti-fibrotic strategies.

## INTRODUCTION

Idiopathic pulmonary fibrosis (IPF) is a chronic and progressive lung disease, which is characterized with fibroblastic proliferation, cystically remodelled air spaces and irregular scars composed of dense collagen fibrosis [1, 2]. Epidemiological investigations have demonstrated that IPF mainly occurs in older individuals over the age of 60 [3], indicating that aging may be one of the major contributors in the pathogenesis of IPF. Aging is a biological process that features decreased control over the internal environment of the body, which could increase the morbidity and mortality of many non-transmissible diseases [4, 5].

In the progression of IPF, the primary target is damage to pulmonary epithelial cells. The pathogenesis of IPF is proposed to occur as the result of repeated and sequential inhaled stimuli-induced injury to lung epithelium [6]. Recently, the expression of p16 and p21 were detected in alveolar epithelial cells derived from IPF lungs, indicating a senescent phenotype in epithelial cells [7]. Increasing evidence has demonstrated an accumulation of senescent cells in IPF lung tissues, which were primarily in the epithelium and less frequently in fibroblasts [7, 8]. In addition, these senescent epithelial cells covering fibroblastic foci were found in IPF lungs, while they were not present in normal lungs [9]. Senescent cells are characterized by stable cell-cycle arrest, resistance to apoptosis and a multifaceted senescence-associated secretory phenotype (SASP), which yields widespread signalling to the external environment [10]. The SASP plays an important role in several physiological processes, such as cellular differentiation during wound repair [11]. Senescent cells could secrete increased amounts of interleukin (IL), including IL-1β, IL-6 and IL-8, which could induce the differentiation of fibroblasts into myofibroblasts [9]. These findings indicated that senescence may exacerbate the pathogenesis of IPF through promoting the abnormal secretory pattern of the lung epithelium and enhancing the resistance of myofibroblasts to apoptosis.

Highly synthetic and α-smooth muscle actin (SMA)-positive myofibroblasts are regarded as the key effector cells of the fibrogenic response during both normal wound healing and pathological fibrosis, including IPF [12]. The persistence of these cells, as a result of a failure in apoptosis, is felt to be a key event in the initiation and progression of fibrosis [13]. Mostly, the increased deposition of extracellular matrix (ECM) found in IPF lungs is due to the activation of fibroblasts in foci. These lesions consist of aggregates of activated fibroblasts that produce excessive ECM within the alveolar space at the site of epithelial cell loss, which do not arise in healthy lungs and highly correlates with survival [14]. However, the underlying mechanisms responsible for the switch to the activated status of fibroblasts remain poorly reported.

It has been reported that key signalling components of the senescence machinery could operate as critical regulators of stem cell function. In cancer cells, a gain of stemness may have profound implications for tumour aggressiveness and clinical outcome [15]. Based on global gene expression profiling, growing evidence has demonstrated that histological poorly differentiated tumours showed preferential overexpression of genes normally enriched in embryonic stem cells (ESCs). The ESC-like transcriptional program activated in different human cancers played a critical role in the prediction of early recurrence, metastasis and poor survival [16–18]. In addition, key regulators of ESC identity, such as Oct4, Sox2 and Nanog, are frequently overexpressed in cancer stem cells derived from different types of cancers [19]. Nanog, as a potential oncogene, has been reported to play a critical role in tumorigenesis. Overexpressed Nanog could predict tumour progression and poor prognosis, which indicates the regulatory role of Nanog in human tumour development [20]. Transfecting cells with Nanog could induce cell transformation [21], suggesting that Nanog might be an essential inducer of stem cell function. In addition, ectopic overexpression of Nanog could enhance the proliferation of NIH3T3 fibroblasts by promoting S-phase entry [21, 22]. However, whether Nanog is a key regulator that induces the uncontrolled migration and proliferation of fibroblasts in the progression of IPF remains unknown.

In our study, we found increased senescence in lung epithelial cells and Nanog-positive fibroblasts in fibrotic lung tissues. *In vitro*, senescent lung epithelial cells could contribute to the activation of pulmonary fibroblasts, along with the aberrant expression of Nanog in pulmonary fibroblasts. Inhibition of cell senescence by rapamycin could effectively impair epithelial cell senescence-induced activation of pulmonary fibroblasts via suppressing the expression of SASP. In addition, we further observed that epithelial cell senescence could promote the binding of β-catenin to the promoter of Nanog, and inhibiting the expression of Nanog could suppress the activation of pulmonary fibroblasts and impair the development of pulmonary fibrosis. Taken together, our work provided a potential strategy for the clinical therapy of IPF through targeting epithelial cell senescence and pulmonary fibroblast activation.

## RESULTS

### Increased epithelial cell senescence occurred in IPF

**Figure 1:**
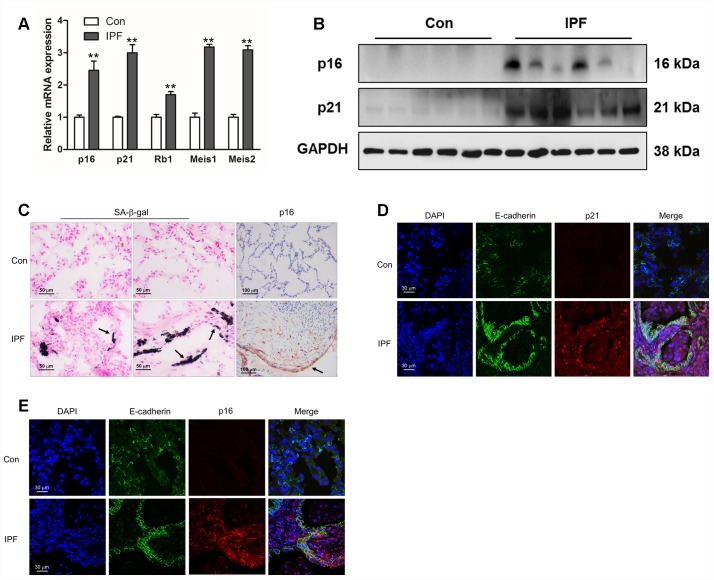
**Increased epithelial cell senescence occurred in idiopathic pulmonary fibrosis (IPF).** (**A**) The mRNA levels of p16 and p21 were measured by Q-PCR, **P < 0.01. (**B**) The protein levels of collagen I, p16 and p21 were measured by Western blot. (**C**) SA-β-gal activity was revealed by X-gal staining. The expression of p16 was determined by immunohistochemical analysis. (**D**, **E**) The normal lung (Con) and lung of a patient with idiopathic pulmonary fibrosis (IPF) were double stained with E-cadherin and p21 (**D**) or p16 (**E**) by immunofluorescence.

As IPF is a senescence-associated disease, the mRNA levels of p16, p21, Rb1, Meis1 and Meis2, typical markers of senescence, were measured by Q-PCR (Figure 1A). The protein levels of p16 and p21 were further determined by Western blot (Figure 1B). The results showed an increased expression of established senescence markers in the lung tissues derived from IPF (Figure 1A and 1B). In addition, we found that increased positive staining of SA-β-gal and p16 were located in the epithelial area (Figure 1C, black arrow), which was hardly seen in normal lung tissues. In order to confirm whether epithelial cells were senescent in the progression of IPF, we performed co-staining on the lung sections of donors and IPF patients. We found that p16 and p21 were elevated in epithelial cells as demonstrated by co-localization with E-cadherin, a marker of epithelial cells (Figure 1D and 1E). These results demonstrated epithelial senescence may be tightly correlated with the development of IPF.

### Rapamycin could protect mice from (bleomycin) BLM-induced pulmonary fibrosis

**Figure 2:**
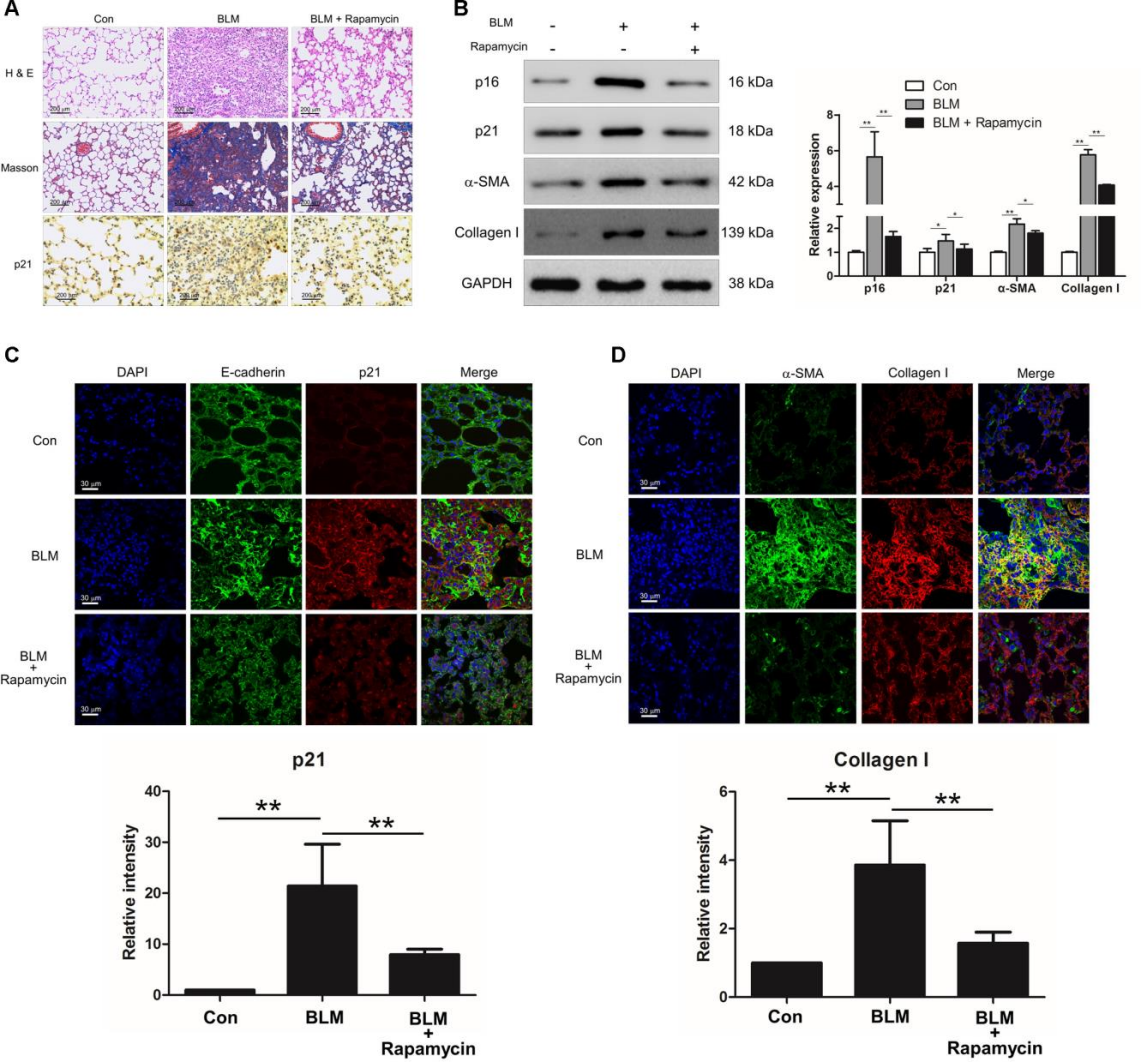
**Rapamycin could protect mice from bleomycin (BLM)-induced pulmonary fibrosis.** Mice (n = 10 in each group) were intraperitoneally injected with vehicle (DMSO/PBS, 10%) or 5 mg/kg rapamycin every other day starting 7 days after administration of BLM (5 mg/kg). (**A**) Pulmonary fibrosis was determined by haematoxylin and eosin (H&E) staining. Collagen was revealed by Masson’s trichrome staining. The expression of p21 was measured by immunohistochemical analysis. (**B**) The protein levels of p16, p21, α-SMA and collagen I were detected by Western blot. The expression levels were quantified with ImageJ (n = 3). GAPDH was used as a loading control, *P < 0.05 and **P < 0.01. (**C**, **D**) The lung tissues were double stained with E-cadherin and p21 (C), α-SMA and collagen I (D) by immunofluorescence. The positive areas of p21 and collagen I were quantified by densitometry (n = 3), **P < 0.01.

We next investigated whether suppression of cell senescence could block the development of pulmonary fibrosis. Experimental animal models were intraperitoneally injected with rapamycin, which is reported as an inhibitor of senescence. Compared with BLM-treated mice, rapamycin evidently decreased the extent of lung lesions and attenuated collagen deposition (Figure 2A) via impairing the expression of senescence markers p16 and p21 (Figure 2B). In addition, the immunofluorescence results also showed that rapamycin profoundly attenuated epithelial cell senescence (Figure 2C) and decreased the levels of fibrotic markers α-SMA and collagen I *in vivo* (Figure 2B and 2D), suggesting that rapamycin could ameliorate BLM-induced pulmonary fibrosis through impairing epithelial cell senescence.

### Epithelial cell senescence could induce pulmonary fibroblast activation via activating Wnt/β-catenin signalling

**Figure 3:**
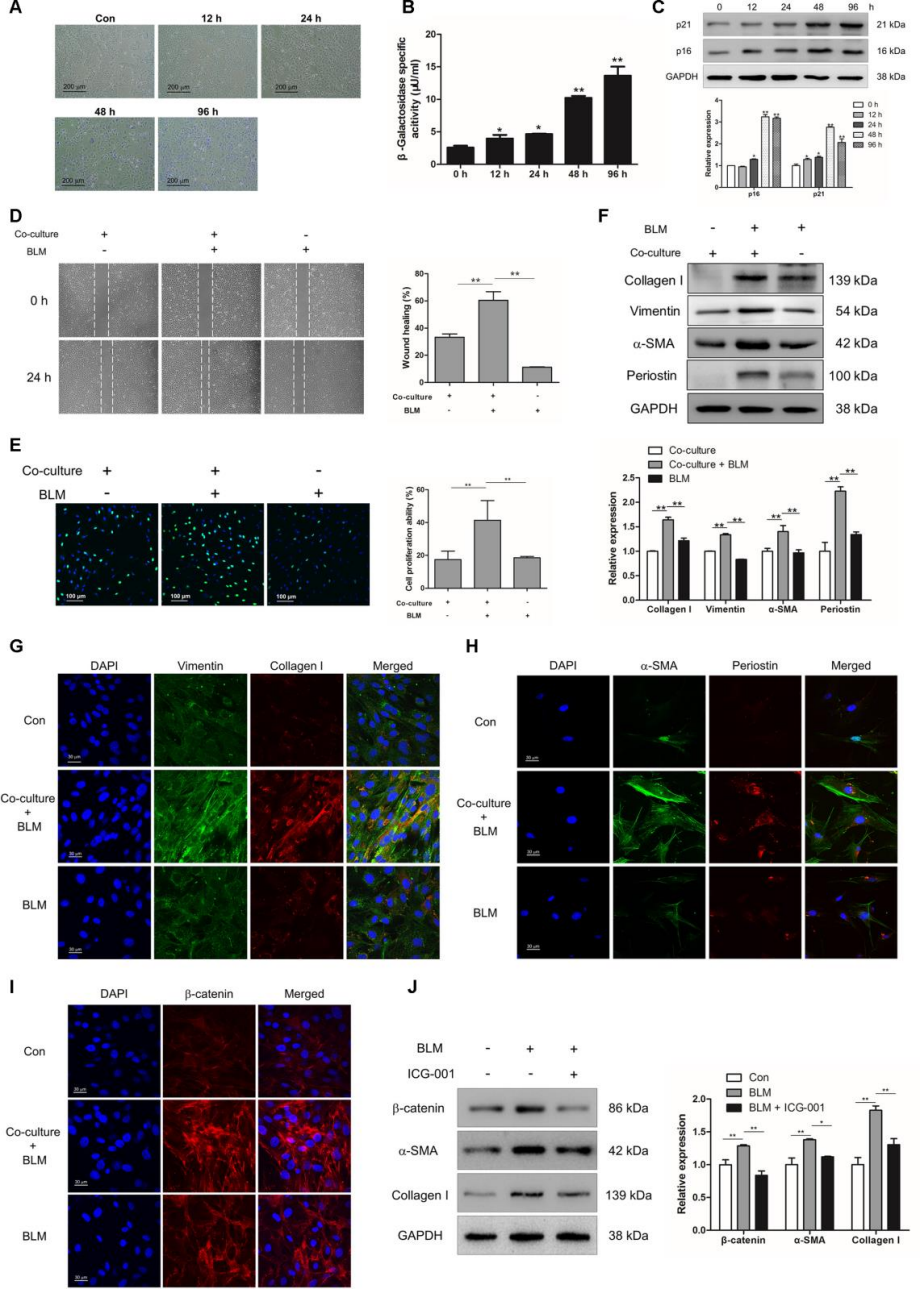
**Epithelial cell senescence could induce pulmonary fibroblast activation via activating Wnt/β-catenin signalling.** (**A**–**C**) MLE-12 cells were treated with bleomycin (BLM, 25 μg/ml) for the indicated times. (A, B) SA-β-gal staining and β-galactosidase activity measurement were performed to detect cellular senescence, * P < 0.05 and ** P < 0.01 vs. 0 h. (**C**) The protein levels of p21 and p16 were measured by Western blot. The expression levels were quantified with ImageJ (n = 3). GAPDH was used as a loading control, *P < 0.05 and **P < 0.01. (**D**–**I**) MLE-12 cells were pre-treated with or without BLM for 3 days. The medium was replaced by fresh medium without BLM and co-cultured with pulmonary fibroblasts for another 3 days. (**D**) The migration capacity of pulmonary fibroblasts was detected by using a wound-healing assay. Wound areas were calculated by ImageJ, **P < 0.01. (**E**) The proliferation ability of pulmonary fibroblasts was measured by EdU assay. The percentage of proliferating cells were calculated by ImageJ, **P < 0.01. (**F**) The protein levels of collagen I, vimentin and α-SMA were determined by Western blot. The expression levels were quantified with ImageJ (n = 3). GAPDH was used as a loading control, **P < 0.01. (**G**) Pulmonary fibroblasts were double stained with vimentin and collagen I by immunofluorescence. (**H**) Pulmonary fibroblasts were double stained with α-SMA and periostin by immunofluorescence. (**I**) The expression of β-catenin was measured by immunofluorescence. (**J**) MLE-12 cells were pre-treated with or without BLM for 3 days. MLE-12 cells were cultured with fresh medium without BLM for another 3 days. The supernatants were collected to culture pulmonary fibroblasts in the presence or absence of ICG-001. The expression of β-catenin, α-SMA and collagen I were examined by Western blot. The expression levels were quantified with ImageJ (n = 3). GAPDH was used as a loading control, *P < 0.05 and **P < 0.01.

In order to uncover the role of epithelial cell senescence in the progression of IPF, we established a BLM- induced epithelial cell senescence model *in vitro*. MLE-12 cells were treated with BLM for 12, 24, 48 and 96 h. BLM could increase the number of senescent cells and enhance the activity of β-galactosidase in a time-dependent manner (Figure 3A and 3B). In addition, the increased expression of p16 and p21 were both increased in a time-dependent manner in BLM-stimulated MLE-12 cells (Figure 3C). To further confirm whether the profibrotic role of senescent epithelial cells in IPF was mediated through inducing the activation of pulmonary fibroblasts, mouse pulmonary fibroblasts were co-cultured with MLE-12 cells that were pre-treated with BLM for 3 days. Interestingly, we found that senescent MLE-12 cells could increase the migration and proliferation of pulmonary fibroblasts (Figure 3D and 3E). In the co-culture system, senescent MLE-12 cells could profoundly induce the expression of α-SMA, vimentin, collagen I and periostin (Figure 3F and 3G). However, the enhanced migration and proliferation of pulmonary fibroblasts did not occur in BLM-treated pulmonary fibroblasts, and BLM treatment could not significantly induce the expression of α-SMA, vimentin, collagen I and periostin (Figure 3D–3H), indicating that BLM could not directly induce the activation of pulmonary fibroblasts, but through inducing the senescence of epithelial cells. In addition, Wnt signalling was activated in the co-culture system, as determined by increased expression of β-catenin in pulmonary fibroblasts (Figure 3I). Inhibition of Wnt signalling via ICG-001 could effectively suppress the expression of α-SMA and collagen I (Figure 3J). These results suggested that senescent epithelial cells could promote the activation of pulmonary fibroblasts via activation of Wnt signalling.

### Rapamycin could suppress epithelial cell senescence and fibroblast activation

**Figure 4:**
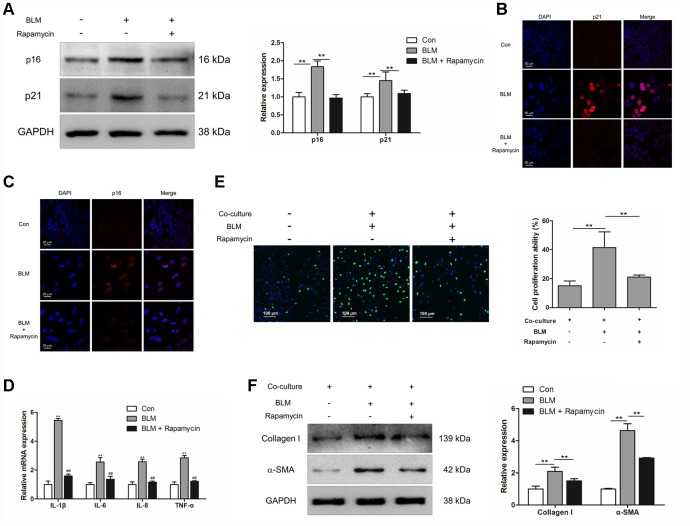
**Rapamycin could suppress epithelial cell senescence and fibroblast activation via impairing the production of SASP.** (**A**–**E**) MLE-12 cells were treated with bleomycin (BLM), followed by treatment with or without rapamycin for 3 days. (**A**) The expression of p16 and p21 were measured by Western blot. The expression levels were quantified with ImageJ (n = 3). GAPDH was used as a loading control, **P < 0.01. (**B**, **C**) The protein levels of p16 and p21 were detected by immunofluorescence. (**D**) The mRNA levels of IL-1β, IL-6, IL-8 and TNF-α were determined by Q-PCR, ** P < 0.01 vs. Con and ## P < 0.01 vs. BLM. (**E**, **F**) MLE-12 cells were treated as in Figure 4A and co-cultured with pulmonary fibroblasts in fresh medium for another 3 days. (**E**) The proliferation ability of pulmonary fibroblasts were measured by EdU assay. The percentage of proliferating cells was calculated by ImageJ, **P < 0.01. (**F**) The expression of α-SMA and collagen I were detected by Western blot. The expression levels were quantified with ImageJ (n = 3). GAPDH was used as a loading control, **P < 0.01.

To further confirm whether inhibition of epithelial cell senescence could suppress the activation of pulmonary fibroblasts, rapamycin (20 nM) was added into the culture medium of MLE-12 cells. Rapamycin treatment could suppress the expression of p16 and p21 in MLE-12 cells (Figure 4A–4C). In addition, we further measured the expression of SASP in MLE-12 cells, including IL-1β, IL-6, IL-8 and TNF-α, which was tightly correlated with pulmonary fibrogenesis [23]. In the process of BLM-induced cell senescence, the mRNA levels of IL-1β, IL-6, IL-8 and TNF-α were robustly increased. Oppositely, dampening cell senescence via rapamycin could result in a decreased expression of IL-1β, IL-6, IL-8 and TNF-α (Figure 4D). Interestingly, in the co-culture system, suppressed epithelial cell senescence also resulted in attenuated proliferation of pulmonary fibroblasts and resulted in decreased expression of α-SMA and collagen I in pulmonary fibroblasts (Figure 4E and 4F). These data indicated that inhibition of pulmonary epithelial cell senescence could impair the activation of pulmonary fibroblasts and therefore the development of pulmonary fibrosis.

### Aberrantly expressed Nanog in activated pulmonary fibroblasts and fibrotic lung tissues were mediated by Wnt/β-catenin

**Figure 5:**
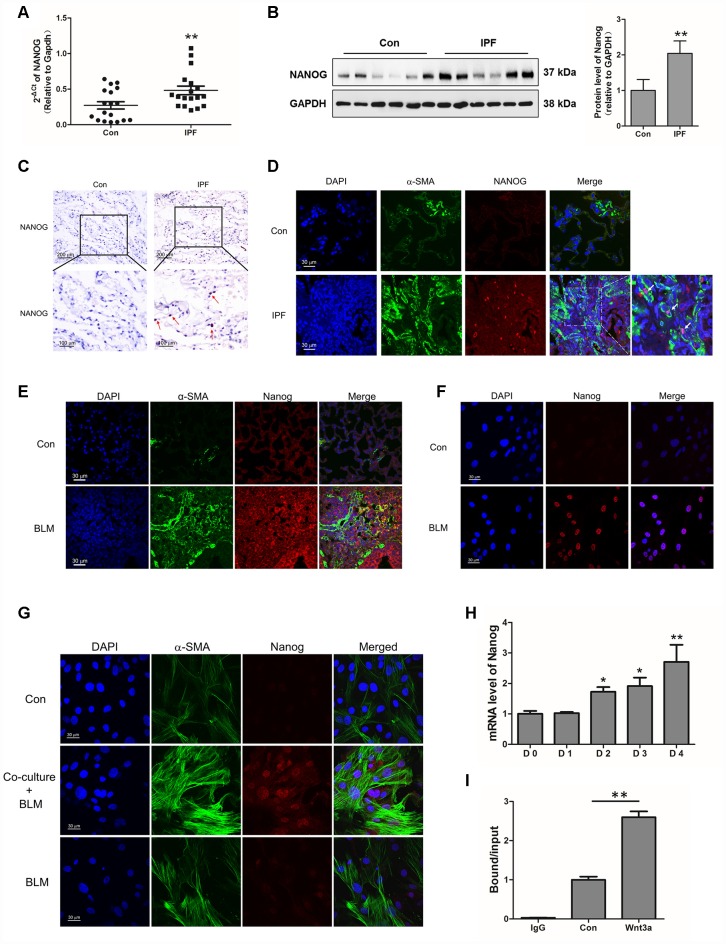
**Aberrantly expressed Nanog in activated pulmonary fibroblasts and fibrotic lung tissues were mediated by Wnt/β-catenin.** (**A**–**C**) The expression of Nanog in lung tissues derived from patients with idiopathic pulmonary fibrosis (IPF) was determined by Q-PCR (**A**), Western blot (**B**) and immunohistochemical analysis (**C**), ** P < 0.01 vs. Con. (**D**) The lung tissues of patients with IPF were double stained with α-SMA and Nanog by immunofluorescence. (**E**) The lung tissues derived from pulmonary fibrosis mouse models were double stained with α-SMA and Nanog via immunofluorescence. (**F**) The expression of Nanog in pulmonary fibroblasts isolated from fibrotic mouse lung tissues were measured by immunofluorescence. (**G**) Cells were treated as in Figure 3D. Pulmonary fibroblasts were double stained with α-SMA and Nanog by immunofluorescence. (**H**, **I**) Pulmonary fibroblasts were treated with Wnt3a for various durations. (**H**) The mRNA level of Nanog was detected by Q-PCR, * P < 0.05 and ** P < 0.01 vs. D0. (**I**) ChIP assays were performed by using chromatin isolated from Wnt3a treated pulmonary fibroblasts. The final DNA extracts were analysed by Q-PCR, ** P < 0.01.

Interestingly, we found that Nanog, a marker of stem cells, was highly elevated in IPF lung tissues (Figure 5A–5C). The co-localization of Nanog and α-SMA also demonstrated that Nanog was overexpressed in the fibroblasts of IPF (Figure 5D), suggesting that the acquired phenotype of Nanog may play an essential role in the activation of pulmonary fibroblasts. In experimental pulmonary fibrosis models, we also found that Nanog staining was increased in fibroblasts as demonstrated by co-localization with α-SMA (Figure 5E). *In vitro*, we further confirmed this finding in pulmonary fibroblasts isolated from mouse fibrotic lung tissues (Figure 5F). In addition, the expression of Nanog was highly elevated in pulmonary fibroblasts co-cultured with senescent epithelial cells (Figure 5G). It has been demonstrated that Wnt signalling plays a critical role in the activation of fibroblasts [24]. In order to further uncover the underlying mechanism between Wnt signalling and Nanog, pulmonary fibroblasts were treated with Wnt3a for various durations. Wnt3a could induce the mRNA expression of Nanog in a time-dependent manner (Figure 5H). In addition, the chromatin immunoprecipitation (ChIP) assay results demonstrated that Wnt3a could enhance the recruitment of β-catenin to the promoter of Nanog, which leads to the aberrant expression of Nanog in pulmonary fibroblasts (Figure 5I). These results indicated that the activation of Wnt/β-catenin signalling is responsible for the acquired phenotype of Nanog in activated pulmonary fibroblasts.

### Nanog silencing could suppress pulmonary fibroblast activation and impair the development of pulmonary fibrosis

**Figure 6:**
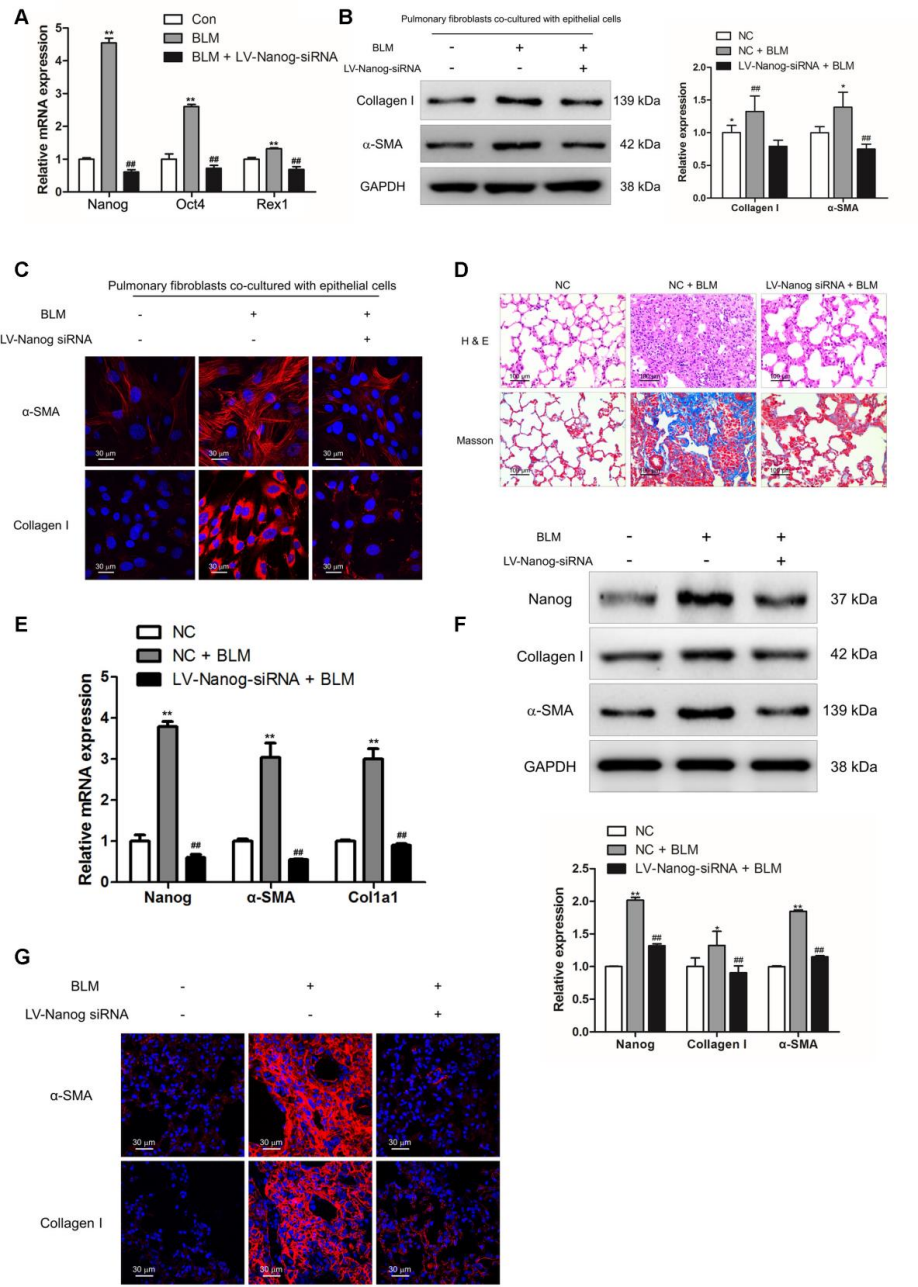
**Nanog silencing could suppress pulmonary fibroblast activation and impair the development of pulmonary fibrosis.** (**A**–**C**) Pulmonary fibroblasts were transfected with LV-Nanog-siRNA and co-cultured with MLE-12 cells as in Figure 3D. (**A**) The mRNA levels of Nanog, Oct4 and Rex1 were measured by Q-PCR, ** P < 0.01 vs. Con and ## P < 0.01 vs. bleomycin (BLM). (**B**) The protein levels of collagen I and α-SMA were determined by Western blot. The expression levels were quantified with ImageJ (n = 3). GAPDH was used as a loading control, *P < 0.05 and **P < 0.01. (**C**) The expression of collagen I and α-SMA were further examined by immunofluorescence staining. (**D**–**G**) Mice were intratracheally injected with 5 × 10^8^ TU/ml LV-Nanog-siRNA or negative control (NC) 7 days after administration of BLM. Mice were sacrificed on day 21 after BLM instillation. (**D**) Pulmonary fibrosis was determined by haematoxylin and eosin (H&E) staining and collagen I was revealed by Sirius Red/Fast Green staining. (**E**) The mRNA levels of Nanog, α-SMA and collagen I were determined by Q-PCR, ** P < 0.01 vs. NC and ## P < 0.01 vs. NC + BLM. (**F**) The protein levels of Nanog, collagen I and α-SMA were measured by Western blot. The expression levels were quantified with ImageJ (n = 3). GAPDH was used as a loading control, *P < 0.05 and **P < 0.01. (**G**) The expression of α-SMA and collagen I were further confirmed by immunofluorescence staining.

In order to verify the role of Nanog in the activation of pulmonary fibroblasts and pulmonary fibrogenesis, pulmonary fibroblasts were transfected with LV-Nanog-siRNA in the co-culture system. LV-Nanog-siRNA treatment could significantly decrease the expression of Nanog in pulmonary fibroblasts, accompanied by the reduction of Oct4 and Rex1, the downstream targets of Nanog (Figure 6A). Inhibition of Nanog have no effect on BLM-induced epithelial cell senescence (Supplementary Figure 1), but attenuate epithelial cell senescence-induced activation of pulmonary fibroblasts, as confirmed by decreased expression of α-SMA and collagen I (Figure 6B and 6C). *In vivo*, intratracheal administration of LV-Nanog-siRNA profoundly decreased pulmonary fibrotic lesions and collagen deposition (Figure 6D).

Suppression of Nanog could effectively attenuate the mRNA and protein levels of collagen I and α-SMA (Figure 6E and 6F). Immunofluorescence staining also revealed that LV-Nanog-siRNA could retard pulmonary fibroblast activation in BLM-induced pulmonary fibrosis, as confirmed by decreased expression of α-SMA and collagen I (Figure 6G). These results demonstrated that inhibition of Nanog could suppress the development of pulmonary fibrosis via impairing the activation of pulmonary fibroblasts.

## DISCUSSION

Fibrosis is a shared pathological characteristic of many fatal lung diseases, such as IPF, a progressive fatal lung disorder related with aging of unknown aetiology [25]. To date, there is no effective cure for these fibrotic diseases, as there is an incomplete understanding of the pathogenesis. In the progression of IPF, epithelial cell senescence has been demonstrated to occur in IPF and experimental lung fibrosis models [26, 27], as confirmed by increased staining for senescence-associated β-galactosidase and p21 specifically in alveolar epithelial cells [9]. Cellular senescence is a physiological programme with defined phenotypic changes in response to replicative or stress-induced telomere shortening. Senescence results in cell cycle arrest, which could prevent the proliferation of damaged cells and promote their elimination via the immune system [28]. In accordance with senescence as a driving factor for pulmonary fibrosis, reduced senescence of alveolar epithelial cells could protect mice from BLM-induced lung damage and fibrosis [29].

In our study, we demonstrated that BLM induced much less fibrosis in rapamycin-treated mice, as assessed by lung collagen content, histopathology and α-SMA expression in the lungs. Rapamycin, as an inhibitor of mTOR signalling, could impair the progression of diseases in different animal models through delaying the entry of cells exposed to chemical stress, ionizing radiation or mitochondrial stress into senescence [30]. Removal of senescent cells could enhance normal tissue function, alleviate the initiation of age-related pathology and attenuate age-induced disorders [31]. Inhibition of cellular senescence by rapamycin could molecularly affect cell cycle arrest, SASP secretion and induction of senescence-associated β-gal [32]. Cell-cell contact between epithelial cells and fibroblasts appears to be essential in signalling cascades and important for wound repair [33]. Pulmonary fibroblasts co-cultured with senescent epithelial cells expressed higher levels of collagen I, vimentin and α-SMA as compared with pulmonary fibroblasts treated with BLM only (Figure 3F), suggesting that senescent epithelial cells could effectively induce the activation of pulmonary fibroblasts. Suppression of epithelial cell senescence via rapamycin could attenuate the activation of pulmonary fibroblasts with decreased expression of SASP, including IL-1β, IL-6, IL-8 and TNF-α, which could induce fibroblast activation [34–36].

Fibroblasts are key effector cells in fibrotic diseases. Upon activation, resting fibroblasts can acquire a myofibroblast phenotype, which is characterized by expression of contractile proteins and enhanced release of ECM [37]. Moreover, fibroblasts in fibrotic foci of IPF also display a phenotype with enhanced proliferation and anti-apoptosis abilities. It was previously reported that a completely chemically defined condition using small molecules could enable efficient and specific reprogramming of mouse fibroblasts into stem-like cells, and acquire long-term self-renew abilities *in vitro* [38]. However, the underlying mechanism between activated pulmonary fibroblasts and stem cell-like reprogramming of fibroblasts in IPF remains unknown. In the lung tissues of IPF patients, we found that Nanog was aberrantly expressed in pulmonary fibroblasts. Nanog is a homeobox-containing transcription factor of approximately 280 amino acids, which functions as a growth-promoting regulator [39]. In the developing mouse embryo, Nanog plays a key role in determining the fate of the inner cell mass (ICM), acting to sustain pluripotency and preventing differentiation [40]. Overexpression of Nanog without any other intervention is sufficient to sustain self-renewal and the anti-apoptosis phenotype [41]. To verify whether Nanog participates in the activation of pulmonary fibroblasts, we established Nanog knock-down experiments to address this issue. We determined that inhibition of Nanog could suppress epithelial cell senescence-induced activation of pulmonary fibroblasts and protect mice from BLM-induced pulmonary fibrosis.

In a previous study, we found Wnt/β-catenin signalling played an essential role in the activation of fibroblasts [42]. Blocking Wnt/β-catenin signalling could suppress fibroblast activation and impair the development of pulmonary fibrosis. In the co-culture system of senescent epithelial cells and pulmonary fibroblasts, we found Wnt/β-catenin signalling was aberrantly activated in pulmonary fibroblasts. Inhibition of Wnt/β-catenin by ICG-001 could affect the induction of Nanog. It was also reported that Wnt/β-catenin signalling played a critical role in maintaining the self-renewal and specific marker expression of cancer stem cells. In the tumour metabolic microenvironment, chronic metabolic stress could cause cancer cells to exhibit cancer stem cell-like properties via activation of Wnt/β-catenin [43], whereas blocking Wnt/β-catenin could effectively suppress cancer stem cell properties [44]. In the canonical Wnt signalling pathway, β-catenin mainly acts as a key signalling transcription factor, which could bind to the promoter areas of Wnt target genes accompanied with Tcf/Lef [45]. It has recently been shown that β-catenin could bind with the promoter of Nanog, thus promoting self-renewal [46]. In this study, we confirmed activation of Wnt signalling could enhance β-catenin binding to the promoter of Nanog in pulmonary fibroblasts.

Taken together, we demonstrated that epithelial cell senescence could induce the activation of pulmonary fibroblasts via increasing the expression of SASP. Inhibition of epithelial cell senescence by rapamycin could effectively suppress the activation of pulmonary fibroblasts and attenuate the development of pulmonary fibrosis. In addition, we further confirmed that epithelial cell senescence could activate Wnt/β-catenin signalling, which mediated the expression Nanog. Suppression of Nanog could impair the activation of pulmonary fibrosis and protect mice from BLM-induced pulmonary fibrosis. Given the importance of cell senescence and Nanog in pulmonary fibrogenesis, our work not only provided an improved understanding of the molecular mechanisms underlying pulmonary fibrosis but also suggested additional targets for therapeutic intervention.

## MATERIALS AND METHODS

### Ethics statement

The animal experiments were performed according to the Guide for the Care and Use of Laboratory Animals (The Ministry of Science and Technology of China, 2006), and all experimental protocols were approved under the animal protocol number SYXK (Su) 2009-0017 by the Animal Care and Use Committee of Nanjing University. All human lung tissues with IPF (n = 6) were obtained from the Department of Lung Transplantation, Wuxi People’s Hospital, Wuxi, China. All diagnoses of IPF were made in accordance with the ATS/ERS criteria for IPF 2011. Normal peripheral tissues (n = 6) from tumour patients supplied by the Thoracic Surgery Department of Nanjing Drum Tower Hospital of the Affiliated Hospital of Nanjing University Medical School were used as controls. The patients who provided normal and IPF lung tissues were male and over 60 years old. Informed consent was obtained from patients, and our study was officially approved by the Ethics Committee of the Medical School of Nanjing University.

### Antibodies

Rabbit monoclonal antibody against β-catenin, mouse polyclonal antibody against α-SMA, rabbit monoclonal antibody against human Nanog, rabbit monoclonal antibody against p16, rabbit monoclonal antibody against p21 and rabbit monoclonal antibody against vimentin were purchased from Abcam (Cambridge, MA). Rabbit monoclonal antibody against mouse Nanog was purchased from Cell Signaling Technology (Cambridge, MA). Mouse monoclonal antibody against collagen I was purchased from Bioss (Beijing, China). Rabbit monoclonal antibody against GAPDH was purchased from BOSTER (Wuhan, China).

### Primary pulmonary fibroblast isolation

Primary pulmonary fibroblasts were isolated as published previously [47]. In brief, mice were sacrificed by cervical dislocation. The lung tissues derived from the mice were cut into 1 mm^3^ fragments and digested with an enzyme mixture containing 2.4 U/ml dispase (Sigma), 0.2% collagenase I (Sigma) and 0.001% DNase (Sigma) for 30 min at 37°C with shaking. Then, the solution was rinsed with 10 ml of warm DMEM/F12 media with 15% FBS three times. The pellet was resuspended in 10 ml of warm DMEM/F12 with 15% FBS and transferred to a 10 cm tissue culture dish that was placed in a tissue culture incubator at 37°C and 5% CO_2_. Fourteen days after the beginning of cell isolation, the cells were harvested and plated on a new plate at 5 x 10^5^ cells/plate EMEM with 15% FBS.

### Induction and treatment of pulmonary fibrosis

All experimental animal procedures were conducted in accordance with humane animal care standards with approval from the Drum Tower Hospital Ethics Committee (Nanjing, China). The animals were acclimated to the environment for 1 week prior to treatment under specific pathogen-free conditions. The mice (n = 6) were administered BLM (Nippon Kayaku, Tokyo, Japan) intratracheally at a dose of 5 mg/kg dissolved in a total of 50 μl sterile saline. The control group was similarly treated with 50 μl of sterile saline.

To explore the role of cell senescence in pulmonary fibrosis, mice were treated with rapamycin (MedChem Express, San Diego, CA), which could protect cells from senescence. Rapamycin (5 mg/kg) or vehicle was injected intraperitoneally every two days from Day 7 to Day 13. Mice were sacrificed at Day 14, and lung tissues were collected for further analysis.

### Establishing co-culture system

An indirect co-culture system was established by using cell culture inserts (0.4 μm PET, 4.5 cm^2^, Millipore). MLE-12 cells were pre-treated with or without BLM accompanied with or without rapamycin for 3 days. In the co-culture system, pulmonary fibroblasts were plated in the lower chamber and MLE-12 cells in the upper chamber, and they were co-cultured for another 3 days. On day 3, the inserts were removed and pulmonary fibroblasts were harvested for EdU assay, wound healing assay, Western blot and immunofluorescence.

### Wound healing assay

After being co-cultured with MLE-12 cells, primary pulmonary fibroblasts were seeded in 6-well plates at a confluence of 80%. The confluent monolayers were wounded as previously described [48]. After washing with PBS, cells were cultured in serum-free medium. Images were captured immediately at 0 h and 24 h. Images were blindly analysed for cell migration ability by ImageJ.

### EdU assay

For EdU assay, the Click-iT EdU Imaging Kit (Life) was used to quantitatively analyse cell proliferation. After being co-cultured with MLE-12 cells, pulmonary fibroblasts were incubated with 10 μM EdU solution for 60 min. EdU incorporated into newly synthesized DNA was further detected by the Alexa Flour 488 azide. The nuclei were stained with DAPI. The images were captured by confocal fluorescence microscope (Olympus). Cell proliferation was analysed by ImageJ.

### Western blot

Samples were lysed by RIPA buffer supplemented with protease inhibitor cocktail on ice. Western blot analysis was performed as previously described [49]. After electrophoresis, proteins were transferred to a PVDF membrane (Millipore). Membranes were incubated with the indicated primary antibodies overnight at 4°C. Horseradish peroxidase-conjugated IgG was used to amplify the signals from the primary antibody. The signals were further detected by using an Odyssey Scanning System (LI-COR, Lincoln, NE).

### Quantitative and reverse transcription PCR

Total RNA was extracted from mouse lung tissues or cultured cells using Trizol reagent (Vazyme, Nanjing, China). The HiScript 1^st^ Strand cDNA Synthesis Kit (Vazyme) was used for reverse transcription PCR (RT-PCR). Comparative quantitative PCR (Q-PCR) was performed by using the SYBR Green Q-PCR Kit (Roche, Germany). Primers are listed in Supplementary Table 1. The Ct values were analysed using the ΔΔCt method and relative changes of mRNA levels were obtained by normalization to GAPDH relative to the control.

### Senescence-associated β-galactosidase assay

The Senescence β-Galactosidase Staining Kit was purchased from Beyotime Biotechnology (Shanghai, China). SA-β-Gal staining was performed according to the manufacturer's protocol. Briefly, cell samples or frozen lung tissue sections were fixed with 4% formaldehyde for 10 min at room temperature. The slides were rinsed with PBS, followed with the incubation of freshly prepared SA-β-Gal staining solution overnight. Then, the cells or tissue sections were washed with PBS at room temperature. To better visualize alveolar structure, the tissue sections were further counterstained with eosin. The images were further captured by using a microscope equipped with a digital camera (Nikon, Tokyo, Japan).

β-galactosidase activity was measured using the β-Gal Activity Assay Kit (BioVision, Beverly, MA) following the manufacturer’s protocol. Briefly, MLE-12 cells were treated with 100 μl ice cold β-Gal assay buffer for 10 min. After centrifugation, the supernatant was reacted with β-Gal substrate in kinetic mode for 30 min at 37°C, and fluorescence (Ex/Em = 480/520 nm) was measured immediately.

### Histopathology

The lung tissues derived from experimental pulmonary fibrosis models were inflated with 4% paraformaldehyde solution overnight and embedded in paraffin before sectioning into 5 μm thick slices. The lung sections were stained with haematoxylin and eosin (H&E) for observation of structure or used for detection of collagen deposition by Masson’s trichrome staining.

### Immunofluorescence

Lung sections or cells were permeabilized with 0.3% Triton X-100 (Sigma) for 5 min and blocked with 5% bovine serum albumin (Sigma) for 1 h. Primary antibodies were applied to lung sections overnight at 4°C. Samples were washed, followed by the incubation of fluorophore-labelled secondary antibody (Invitrogen) for 1 h at 37°C. Nuclei were stained with DAPI (Sigma). The images were captured by confocal fluorescence microscope (Olympus, Tokyo, Japan).

### Immunohistochemistry

Paraformaldehyde-fixed and paraffin-embedded 5 μm sections of mouse lung were deparaffinized in xylene, rehydrated in alcohols and pre-treated in citrate buffer for 30 min at 100°C for epitope-antigen retrieval; endogenous peroxidase activity was quenched with 3% H_2_O_2_ for 10 min. Tissue sections were blocked with 3% bovine serum albumin for 1 h at 37°C. Tissue sections were further incubated with the indicated primary antibodies overnight at 4°C. Then, the Polink-2 plus Polymer HRP Detection System (ZSGB-BIO, Beijing, China) was applied for positive staining. Slides were photographed on the Nikon Eclipse-50i microscope system (Tokyo, Japan).

### Chromatin immunoprecipitation (ChIP)

Cells for ChIP were cultured in 10 × 10 cm dishes. The ChIP assay was performed by following the instructions of the Pierce Agarose ChIP Kit (Thermo Scientific). Pulmonary fibroblasts were cross-linked with 1% formaldehyde, followed by 0.1 M glycine to stop the reaction. Then, the chromatin was sheared into fragments of 500–1000 bp in length and the DNA-protein complex of chromatin fragments was precipitated by anti-β-catenin or anti-IgG antibody. The DNA was then eluted and extracted with phenol-chloroform and amplified by Q-PCR. Axin2 promoter-specific primers were used to amplify the β-catenin binding regions. The primers were as follows: Axin2 promoter sense, 5'-TCATCTGAACCTCCTCTC-3', Axin2 promoter antisense, 5'-GTTGCTTGATTTGAATTTGAG-3'.

### Co-immunoprecipitation (Co-IP) and protein analysis

Co-IP was performed using the Pierce Co-IP Kit from Thermo Scientific following the manufacturer’s protocol. Briefly, the FoxM1 antibody (Santa Cruz) was first immobilized for 2 h using AminoLink Plus Coupling Resin. The resin was then washed and incubated with the lysate of lung tissues overnight. A negative control that was provided with the IP kit to assess nonspecific binding received the same treatment as the Co-IP samples. Immunocomplexes were washed five times with washing buffer before being resolved by SDS-PAGE and immunoblotted with the indicated antibodies.

### Statistical analysis

Statistical analysis was performed using Prism 5 (GraphPad Software, La Jolla, CA). The data are presented as means ± SDs. Differences were analysed for significance (P < 0.05) by one-way ANOVA using SPASS for Windows version 11.0 (SPASS, Chicago, IL).

## Supplementary Material

Supplementary Figure 1

Supplementary Table 1
