# Supplementary material for: Epithelial cell senescence induces pulmonary fibrosis through Nanog-mediated fibroblast activation
Source: Aging (Albany NY). 2019 Dec 31;12(1):242–59. doi: 10.18632/aging.102613 (PMC6977687; doi:10.18632/aging.102613)
Supplement: Supplementary Figure 1 [file aging-12-102613-s002..pdf]

SUPPLEMENTARY FIGURE

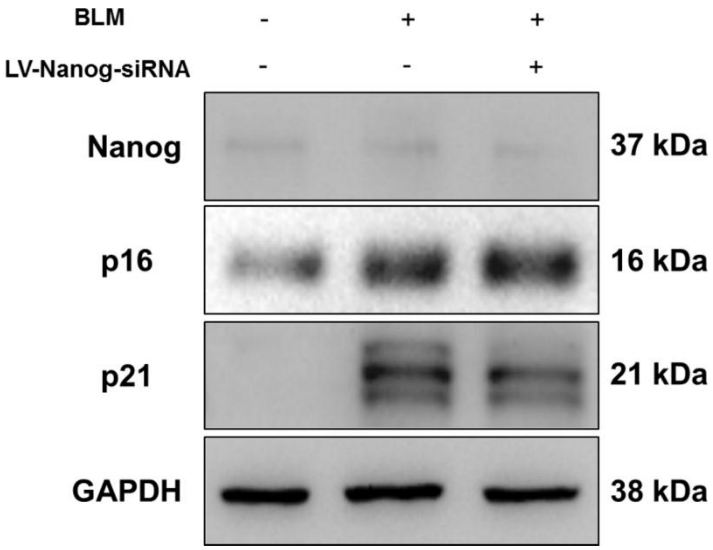

Supplementary Figure 1. Inhibition Nanog has no effect on bleomycin (BLM)-induced epithelial cell senescence. MLE-12 cells were transfected with LV-Nanog-siRNA in the presence or absence of BLM. The expression of Nanog, p16 and p21 were measured by Western blot.
