# Supplementary material for: Epithelial cell senescence induces pulmonary fibrosis through Nanog-mediated fibroblast activation
Source: Aging (Albany NY). 2019 Dec 31;12(1):242–59. doi: 10.18632/aging.102613 (PMC6977687; doi:10.18632/aging.102613)
Supplement: Supplementary Table 1 [file aging-12-102613-s001..pdf]

SUPPLEMENTARY TABLE

Supplementary Table 1. RT-PCR primers.

Gene names	Sense	Antisense
Human-p16	AGGGCTTCCTGGACACGCTGGTGGT	CGGCATCTATGCGGGCATGGTTA
Human-p21	TGATTAGCAGCGGAACAAGGAGT	TGGAGAAACGGGAACCAGGACAC
Human-Rb1	CTCAGATTCACCTTTATTTGAT	CAGACAGAAGGCGTTCACAAAGT
Human-Meis1	GACCAGAAGAAGACAGAGCGGATGA	CGCCCGAGTCACTGAGCATAAAA
Human-Meis2	GTCTGCTCCTCCGACTCCTTCAA	ATCGCACAGTTCGTGGACCTTTT
Human-Nanog	ACCTATGCCTGTGATTTGTGGG	AGAAGTGGGTTGTTTGCCTTTG
Human-GAPDH	GAATGGGCAGCCGTTAGGAAAGC	AGCATCACCCGGAGGAGAAATCG
Mouse-Nanog	CCTGATTCTTCTACCAGTCCCAAAC	CACAGTCCGCATCTTCTGCTTCC
Mouse-IL1β	CAAGCAATACCCAAAGAAGAAGA	ATTAGAAACAGTCCAGCCCATAC
Mouse-IL-6	GGAGCCCACCAAGAACGATAGTCAA	GTCACCAGCATCAGTCCCAAGAA
Mouse-IL-8	GGCTTTGCGTTGATTCTGGGAACT	AGCGGTGTCCTGATTATCGTCCT
Mouse-TNF-α	CATTTGAGGACGATAATCAGGAC	AAAGCAGAAAGACTAAGCAGGAA
Mouse-α-SMA	CCCAGATTATGTTTGAGACCTTC	ATCTCCAGAGTCCAGCACAATAC
Mouse-Oct4	TTCCCTCTGTTCCCGTCACTGCT	GTCTACCTCCCTTGCCTTGGCTC
Mouse-Rex1	AGGGAGAATGGTGCTCTGGGTCA	TGTCATCGTCATCTATGCTTGTGGC
Mouse-Col1a1	CTTCTGGTCCTCGTGGTCTCCCT	AAGCCTCGGTGTCCCTTCATTCC
Mouse-GAPDH	AGGTCGGTGTGAACGGATTTG	TGTAGACCATGTAGTTGAGGTCA
